# An Ultrasensitive Norfentanyl Sensor Based on a Carbon Nanotube-Based Field-Effect Transistor for the Detection of Fentanyl Exposure

**DOI:** 10.1021/acsami.3c05958

**Published:** 2023-07-31

**Authors:** Wenting Shao, Zidao Zeng, Alexander Star

**Affiliations:** † Department of Chemistry, University of Pittsburgh, Pittsburgh, Pennsylvania 15260, United States; ‡ Department of Bioengineering, University of Pittsburgh, Pittsburgh, Pennsylvania 15261, United States

**Keywords:** carbon nanotube, field-effect transistor, biosensor, opioid, fentanyl overdose, norfentanyl

## Abstract

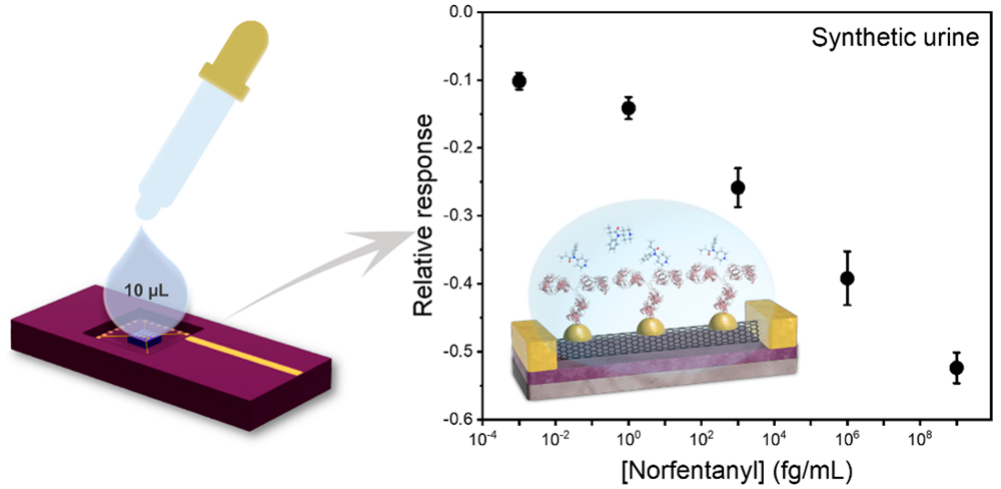

The opioid crisis is a worldwide public health crisis that has affected millions of people. In recent years, synthetic opioids, primarily illicit fentanyl, have become the primary driver of overdose deaths. There is a great need for a highly sensitive, portable, and inexpensive analytical tool that can quickly indicate the presence and relative threat of fentanyl. In this work, we develop a semiconductor enriched (sc-) single-walled carbon nanotube (SWCNT)-based field-effect transistor (FET) biosensor functionalized with norfentanyl antibodies for the sensitive detection of norfentanyl, the primary inactive metabolite of fentanyl, in urine samples. Different sensor configurations were explored in order to obtain the most optimized sensing results. Moreover, by employing the “reduced” antibody, we achieved orientated immobilization of the norfentanyl antibody and thus brought the antigen–antibody interaction closer to the sensor surface, further improving the sensitivity. The reported norfentanyl biosensors have a limit of detection in the fg/mL region in both calibration samples and synthetic urine samples, showing ultrasensitivity and high reliability.

## Introduction

Fentanyl (*N*-phenyl-N-[1-(2-phenylethyl)piperidinyl]-propanamide) is a potent synthetic opioid that is used as a pain reliever and an anesthetic. It is approximately 50–100 times more potent than morphine.^1^ However, due to its pharmacological effects, the overdose of fentanyl can cause difficulties in breathing and can lead to death. According to the U.S. Centers for Disease Control and Prevention (CDC), synthetic opioids are the primary driver of overdose deaths in the United States, making opioid overdose deaths a major public health crisis.^2,3^ At the same time, fentanyl can also be found in combination with other drugs such as heroin or cocaine. Therefore, there is a great need for a highly sensitive, portable, and inexpensive analytical technique that can quickly indicate the presence and relative threat of fentanyl.

In human body, fentanyl gets rapidly metabolized in the liver to norfentanyl via oxidative N-dealkylation and to 4-anilino-N-phenethylpiperidine (4-ANPP) via hydrolysis.^4−6^ Norfentanyl, as the primary inactive metabolite of fentanyl, can also be detected in body fluids, such as urine and blood, and is sometimes tested for in people who have been prescribed fentanyl or other opioids or in individuals who may have used or been exposed to these drugs. Testing for norfentanyl can be more reliable and accurate, particularly in cases where the sample may be degraded or the pH of the urine is not optimal. In addition, norfentanyl has a longer detection window, thus testing for norfentanyl can provide more information about an individual’s past exposure to fentanyl.^7,8^

Instrumental analytical methods, such as gas chromatography–mass spectrometry (GC-MS) and liquid chromatography-tandem mass spectrometry (LC-MS/MS) are the most commonly used techniques for the detection of norfentanyl.^9−15^ Laboratories often combine chromatographic methods with immunoassays to ensure both high sensitivity and specificity.^16,17^ However, these techniques require specialized equipment and training, making them relatively expensive and time-consuming. Meanwhile, due to the lack of redox activity of norfentanyl, certain electrochemical methods, although are generally considered to be rapid, simple, and low-cost, are not suitable for norfentanyl detection.^18,19^

Carbon nanomaterial-based field-effect transistor (FET) biosensors have shown remarkable sensitivity and low detection limits for a variety of biological analytes.^20−25^ Previously, an aptamer-based graphene FET (AptG-FET) platform was reported for the simultaneous detection of three different opioid metabolites in wastewater.^26^ The AptG-FET platform with a coplanar Pt gate enabled multianalyte detection on a single chip and achieved a picogram per milliliter level limit of detection for noroxycodone (NX), 2-ethylidene-1,5- dimethyl-3,3-diphenylpyrrolidine (EDDP), and norfentanyl. Semiconductor-enriched (sc-) single-walled carbon nanotubes (SWCNTs), on the other hand, are particularly promising candidates for FET biosensors because the high-purity semiconducting content offers high on/off ratio for FETs,^27^ facilitating ultrasensitivity of sc-SWCNT-based FET biosensors.^28,29^ Additionally, due to the presence of functional groups on the sidewalls, SWCNTs provide more avenues for functionalization with custom-designed chemistry to preferentially interact with target biomolecules, which leads to excellent sensitivity in complex media, such as saliva,^30^ sweat,^31^ and serum.^32^

This work aims to develop a functionalized carbon nanotube-based biosensor for ultrasensitive norfentanyl detection in body fluids and consequently make time-sensitive decisions. Here, we report a norfentanyl antibody-functionalized sc-SWCNT-based FET biosensor that achieved fg/mL level limit of detection of norfentanyl in both calibration samples and synthetic urine samples. To optimize the detection in the sensing matrix, we explored different approaches for the attachment of antibody on sc-SWCNTs, namely, the direct coupling approach and gold nanoparticle (AuNP) approach, and different biorecognition elements for the detection of norfentanyl. Distinct sensing behaviors were observed during the sensing experiments, implying different sensing mechanisms with different sensor configurations. By analyzing the sensing performances, we found that the AuNP-decorated sc-SWCNT FET biosensors provide a more robust platform for antibody functionalization and are less susceptible to nonspecific species present in biological samples, making them good candidate for point-of-care tool for the detection of fentanyl exposure. Moreover, we successfully applied our sensor fabrication with a flexible FET electrode and demonstrated good sensing performances for norfentanyl detection with a portable sensing setup.

## Results and Discussion

**Figure 1:**
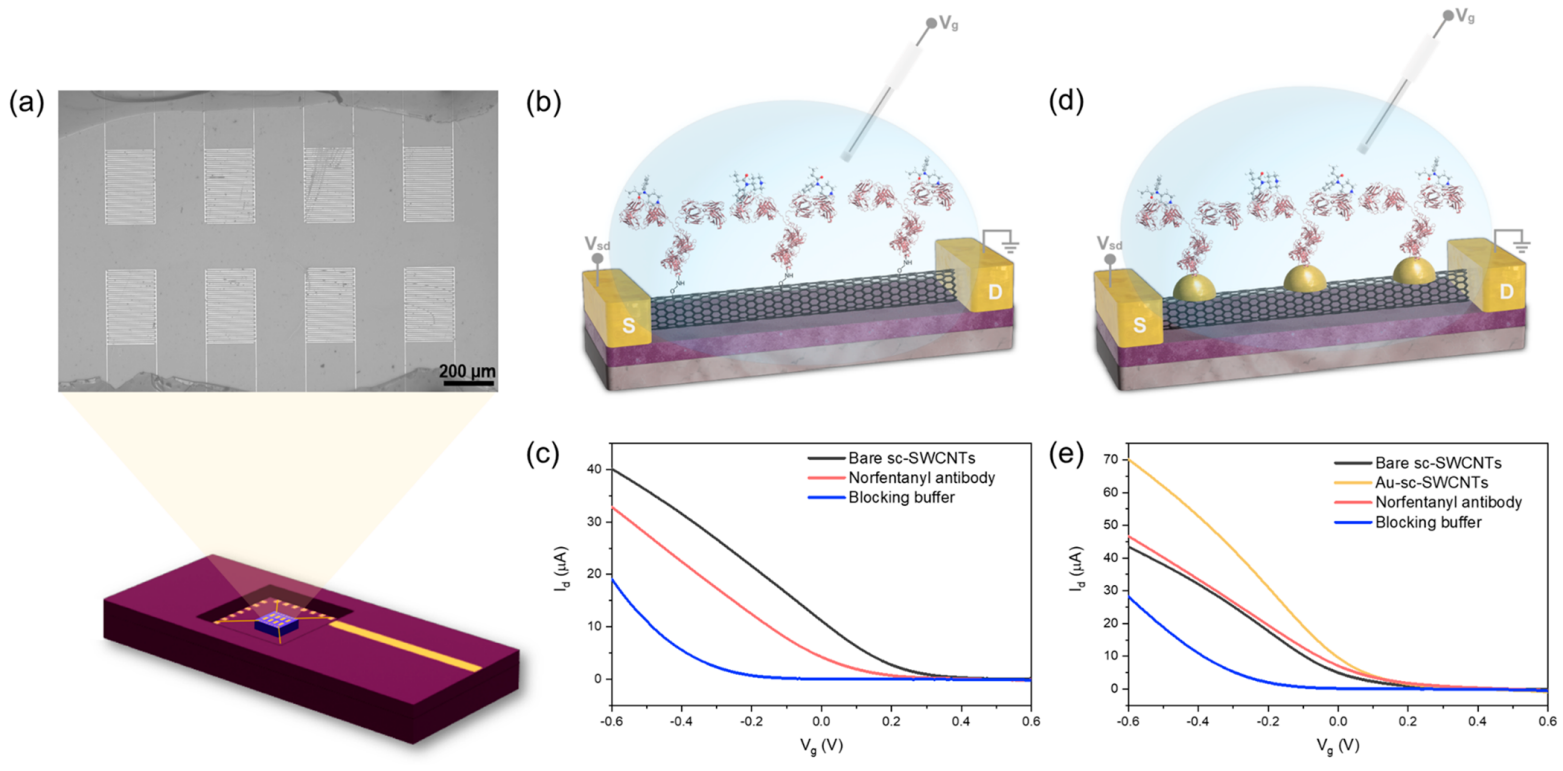
Norfentanyl antibody functionalized SWCNT-based FET biosensor. (a) Top: Optical image of the sensing chip with 8 devices. Bottom: The sensing chip was wire-bonded in a package for measurements. (b) Schematic illustration of a norfentanyl antibody functionalized SWCNT-based FET biosensor via direct coupling approach. (S: Source; D: Drain.) (c) FET transfer characteristics of each functionalization step using a direct coupling approach. (d) Schematic illustration of a norfentanyl antibody functionalized SWCNT-based FET biosensor via the AuNP approach. (e) FET transfer characteristics of each functionalization step using the AuNP approach.

As shown in Figure 1a, the sensor chip contains eight devices with interdigitated gold source and drain electrodes and is packaged in a chip carrier for FET measurements. The FET channel length, i.e., the interdigital gap, is 10 μm. Semiconductor-enriched (sc-) single-walled carbon nanotubes (SWCNTs) were deposited between interdigitated gold electrodes via dielectrophoresis (DEP), providing conducting channels, as well as a platform for antibody immobilization.

The attachment of antibody on the sc-SWCNT FET devices was achieved through two different approaches: a direct coupling approach (Figure 1b) and a gold nanoparticle (AuNP) approach (Figure 1d). For direct coupling approach, norfentanyl antibodies were immobilized on sc-SWCNTs through covalent chemical bonds by EDC/NHS coupling between the carboxylic acid groups on the sidewalls of carbon nanotubes and the free amine groups on the antibodies. AuNP approach utilized the surface of AuNPs decorated on the sc-SWCNTs for the binding of norfentanyl antibodies. For both types of FET devices, changes of the chemical environment of the sc-SWCNTs during each step of the functionalization process were reflected in the changes in the FET transfer characteristics. The direct coupling of norfentanyl antibodies induces a shift of threshold voltage toward more negative gate voltages and a decrease in the device conductance in the FET characteristics, and a further shift of the threshold voltage and decrease in the conductance can be observed after the blocking buffer was applied (Figure 1c). For Au-sc-SWCNT FET devices, while the decoration of AuNPs improves the conductivity of the sc-SWCNTs, yielding higher source-drain current in the p-type region, the attachment of antibodies on the AuNPs lowers the device conductance, and a similar negative shift of the threshold voltage and decrease in the conductance is observed in the *I*_d_–*V*_g_ curve after the addition of the blocking buffer (Figure 1e).

**Figure 2:**
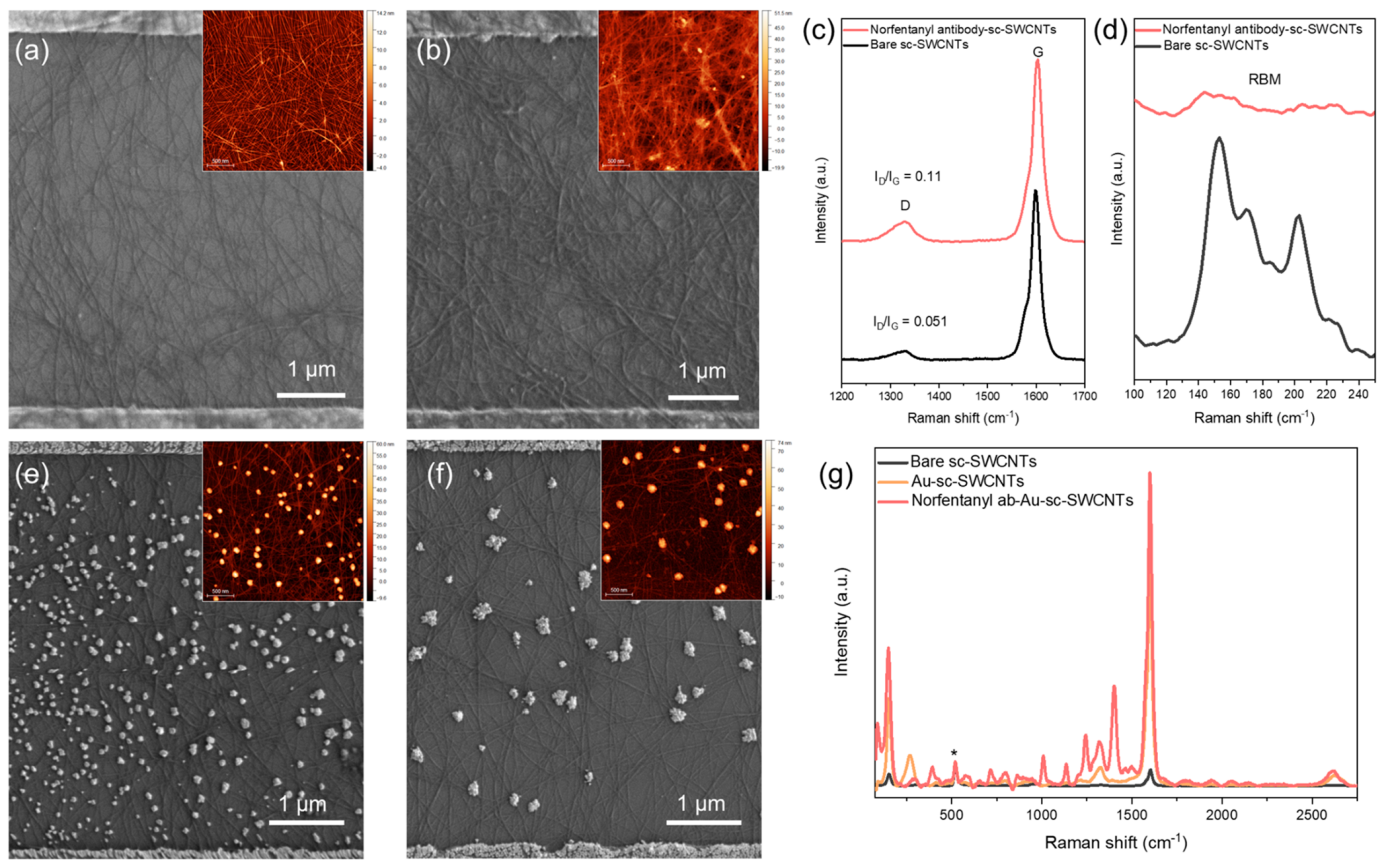
Device characterizations. (a) SEM image and AFM image (inset) of SWCNT networks deposited between source and drain electrodes on a FET device. (Inset scale bar: 500 nm) (b) SEM image and AFM image (inset) of SWCNT networks after norfentanyl antibody functionalization. (Inset scale bar: 500 nm) (c) D and G peak regions of Raman spectra of the SWCNTs before and after the immobilization of norfentanyl antibody. The Raman spectra were recorded using a 638 nm excitation laser. All spectra were normalized to the G peak at 1587 cm^–1^. (d) Radial breathing mode (RBM) regions of Raman spectra were recorded using a 785 nm excitation laser. All spectra were normalized to the Si peak at 507 cm^–1^ (not shown). (e) SEM image and AFM image (inset) of Au-sc-SWCNTs and (f) norfentanyl ab-Au-sc-SWCNTs (inset scale bar: 500 nm). (g) Raman spectra of the FET device during each functionalization step using the AuNP approach. The Raman spectra were recorded using a 638 nm excitation laser. All spectra were normalized to the Si peak at 507 cm^–1^ (denoted by the asterisk).

As shown in Figure 2a, the deposition of sc-SWCNTs created a dense network of carbon nanotubes between the interdigitated electrodes, which was characterized by both scanning electron microscopy (SEM) and atomic force microscopy (AFM). The average diameter of the sc-SWCNT bundles is 3.4 ± 1.1 nm. The covalent attachment of the norfentanyl antibody was evidenced by the thickening of SWCNT strands with small nucleation of the antibody on the SWCNT surfaces (Figure 2b). Additionally, the appearance of the N 1s peak in X-ray photoelectron spectroscopy also confirms the successful immobilization of antibodies on sc-SWCNTs (Figure S1). Raman spectroscopy was utilized to understand the effect of antibody functionalization on the structure of SWCNTs. Although no prominent peaks from the antibody were observed after the direct coupling of the antibodies, the analysis of the D and G features from sc-SWCNTs reveals an increase in the *I*_D_/*I*_G_ ratio from 0.051 to 0.11, suggesting an increase in the degree of functionalization of SWCNTs, likely due to the covalent bonding of antibodies to the SWCNTs (Figure 2c). The intensity of the radial breathing mode (RBM) was also drastically reduced due to the functionalization on the sidewalls of sc-SWCNTs (Figure 2d).^33,34^

When norfentanyl antibodies were introduced to AuNP-decorated SWCNTs, the antibodies predominantly bound to the AuNP surfaces (Figure 2e and f). Average height of the AuNPs rose from 48.9 ± 7.5 nm to 59.5 ± 7.3 nm by AFM characterizations. The 10.6 nm increase in height matched the size of IgG type antibodies.^35^ The decoration of AuNPs on sc-SWCNTs also creates a substrate for surface enhanced Raman scattering (SERS).^36,37^ Raman intensity of sc-SWCNTs increased about 20 times due to the SERS effect. More importantly, Raman features from the norfentanyl antibody, which are generally hard to resolve at low concentration, appear in the Raman spectra (Figure 2g), indicative of the successful immobilization of antibodies on sc-SWCNTs.

While fentanyl, as a redox active molecule, can be detected using electrochemical methods such as cyclic voltammetry, differential pulse voltammetry (DPV), and square wave voltammetry (SWV), no redox activity was found for norfentanyl when we conducted cyclic voltammetry studies with norfentanyl using sc-SWCNTs as the working electrode (Figure S2). The norfentanyl antibody-functionalized sc-SWCNT FET biosensor responses were investigated by employing a liquid-gate FET configuration and recording FET transfer characteristics (*I*_d_–*V*_g_), from which rich information about the biorecognition process, sensing mechanism, and sensing performances can be extracted. The FET transfer characteristics were measured using a portable dual-channel potentiostat to enable norfentanyl detection on site. Standard resistor tests suggest that no significant difference is observable between the portable potentiostat and laboratory high precision sourcemeters (Figure S3). The latter were used in our previous SWCNT-based FET biosensor work.^38−40^ Moreover, it is worth mentioning that albeit direct FET measurements in norfentanyl samples simplify the sensing procedure and facilitate real-time sensing, the high ionic strength of phosphate buffered saline (PBS) or synthetic urine causes Debye screening and lowers sensitivity (Figure S4). As a result, all FET characteristics were recorded in 0.001× PBS after sample incubation to overcome the Debye screening limitation.

**Figure 3:**
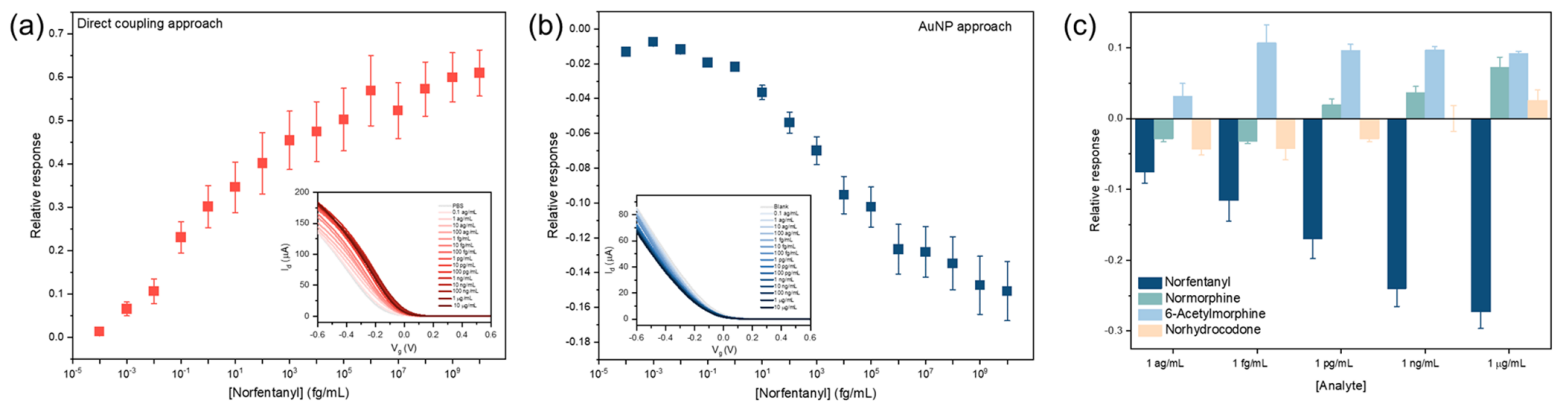
Norfentanyl sensing in PBS. (a) Norfentanyl sensing performance using ab-sc-SWCNT devices (direct coupling approach). Inset shows FET transfer characteristics of norfentanyl ab-sc-SWCNT devices upon adding increasing concentrations of norfentanyl. (b) Norfentanyl sensing performance using norfentanyl ab-Au-sc-SWCNT devices (AuNP approach). Inset shows FET transfer characteristics of norfentanyl ab-Au-sc-SWCNT devices upon adding increasing concentrations of norfentanyl. (c) Control experiments with nonspecific drug metabolites using norfentanyl ab-Au-sc-SWCNT devices.

The sensor responses at different norfentanyl concentrations are plotted in Figure 3a and b, from which we evaluated the sensor performance of each type of sensor. Sensors adopting the direct coupling approach had a dynamic range of 100 ag/mL to 100 fg/mL, calibration sensitivity of 0.069, and limit of detection (LOD) of 2.0 fg/mL. AuNP-decorated sensors had a dynamic range of 100 fg/mL to 100 pg/mL, calibration sensitivity of 0.021, and LOD of 3.7 fg/mL (Figure S5). The calibration curve also provides information about the binding affinity between norfentanyl and its antibody at the sensor interface. The dissociation constant (*K*_d_), which corresponds to the concentration at half-maximum response, is 2.8 fg/mL for sensors adopting the direct coupling approach and 2.0 pg/mL for AuNP-decorated sensors.^41^

Despite both types of devices demonstrating sensing capabilities toward norfentanyl with an LOD in the fg/mL region, the calibration curves displayed opposite trends upon norfentanyl exposure. For devices adopting the direct coupling approach, the binding of norfentanyl on the device surface induced an increase in the conductance of the FET device, resulting in a corresponding increase in the relative response. This result is consistent with what we previously observed with cortisol antibody-functionalized sc-SWCNT FET biosensors for cortisol sensing utilizing the same approach.^31^ Similarly, the binding between norfentanyl and norfentanyl antibody likely relies on the nonpolar hydrogen−π interaction and cationic-π interaction between norfentanyl and amino acid residues inside the binding sites of the antibody such as aspartate (Asp) and tyrosine (Try).^42^ Therefore, we attributed the sensor response to the redistribution of charges on the antibody upon norfentanyl binding, making the antibody less positively charged and consequently p-doping the sc-SWCNTs.

When decorated with AuNPs, norfentanyl antibodies predominantly bound to the AuNP surfaces; thus, the AuNP-nanotube interface is most responsible for the sensing. At the junction of metal nanoparticles and semiconductors, Schottky barriers form. Upon binding of norfentanyl, the charge redistribution of the norfentanyl antibody lowers the work function of the AuNPs, increasing the Schottky barrier, and as a result, a decrease in the conductance was observed in the experiment.^43^

The specificity of both types of sensors was investigated using sc-SWCNT FET devices without the conjugation of norfentanyl antibodies. The lack of sensor responses when norfentanyl antibodies were absent indicated that the sensors have high specificity (Figure S6). In terms of selectivity, other opioid metabolites, namely, normorphine (the metabolite of morphine), norhydrocodone (the metabolite of hydrocodone), and 6-acetylmorphine (indicative of heroin use), were added to the norfentanyl ab-Au-sc-SWCNT FET biosensors in the same concentration range as norfentanyl. However, the sensor behaviors are markedly different (Figure 3c), further confirming that the observed sensor response with norfentanyl is associated with the specific interaction between norfentanyl and its antibody, allowing for good selectivity toward norfentanyl.

**Figure 4:**
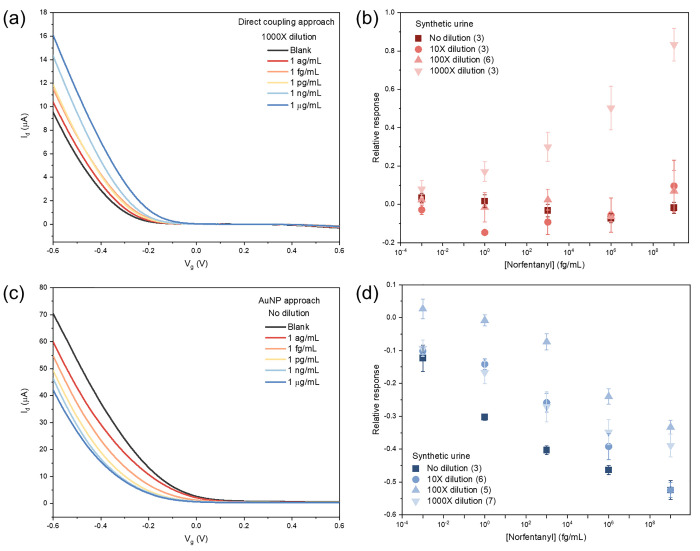
Norfentanyl sensing in synthetic urine. Direct coupling approach: (a) FET characteristic curves for norfentanyl sensing in 1000× diluted synthetic urine using norfentanyl ab-sc-SWCNT devices; (b) Calibration plot of the devices for norfentanyl sensing in different dilutions of synthetic urine. AuNP approach: (c) FET characteristic curves for norfentanyl sensing in synthetic urine without dilution using norfentanyl ab-Au-sc-SWCNT devices; (d) calibration plot of the devices for norfentanyl sensing in different dilutions of synthetic urine. All data points plotted in the calibration plots are the mean ± standard error (SE). The number of devices (n) used for calculation are indicated in the parentheses in the legend.

In the interest of studying past fentanyl exposure of an individual, we conducted norfentanyl sensing experiments in synthetic urine with both types of antibody-functionalized FET biosensors. The norfentanyl containing synthetic urine samples were also diluted 10-, 100-, and 1000-fold in PBS to investigate the susceptibility of these sensors to interferences. For FET devices fabricated via the direct coupling approach, the sensitivities of the devices are drastically reduced in nondiluted, 10-fold and 100-fold diluted synthetic urine samples, and the norfentanyl sensing capability is only observed in 1000-fold diluted samples (Figure 4a and b). However, sensors with norfentanyl antibodies immobilized on AuNPs demonstrated similar norfentanyl sensing behavior regardless of the extent of dilution (Figure 4c and d).

The compromised sensitivity of the sensors fabricated via the direct coupling approach is likely due to the interactions between the nonspecific species present in the synthetic urine with the carbon nanotubes. By immersing the FET devices in 1× synthetic urine without norfentanyl for 10 min, it is evident that the nonspecific species in the synthetic urine can alter the FET characteristics of both types of devices (Figure S7a and b). It is less likely that the antigen–antibody interaction is impaired because of two reasons: (1) the ionic strength of the synthetic urine is similar to that of PBS, which is optimal for antigen–antibody binding; and (2) while high concentration of urea (∼6 M) can be used for the dissociation of antigen-bound antibody, the urea concentration is relatively low (∼250 mM) in synthetic urine.^44,45^ With a higher extent of dilution, the interactions between the interfering species and the carbon nanotubes became negligible, and the specific sensor response restores. On the opposing side, for devices decorated with AuNPs, fewer carbon nanotube surfaces are exposed to the biological environment, therefore demonstrating less susceptibility to interferences.

Interestingly, both types of sensors respond to the nonspecific synthetic urine components in a similar way as to norfentanyl (Figure S7c). As a result, a collective effect of the specific and nonspecific interactions leads to an increase in the calibration sensitivity of both sensors in 1000-fold diluted synthetic urine. The comparison of the sensor performances in PBS and 1000-fold diluted synthetic urine reveals a 55.6% rise in calibration sensitivity for devices with directly coupled antibodies and a 55.2% rise in calibration sensitivity for devices decorated with AuNPs (Figure S8). However, in order to improve the quantitative ability of the sensors, in real-life practices, background elimination should be considered when applying this biosensor for the detection of norfentanyl in clinical samples.

**Figure 5:**
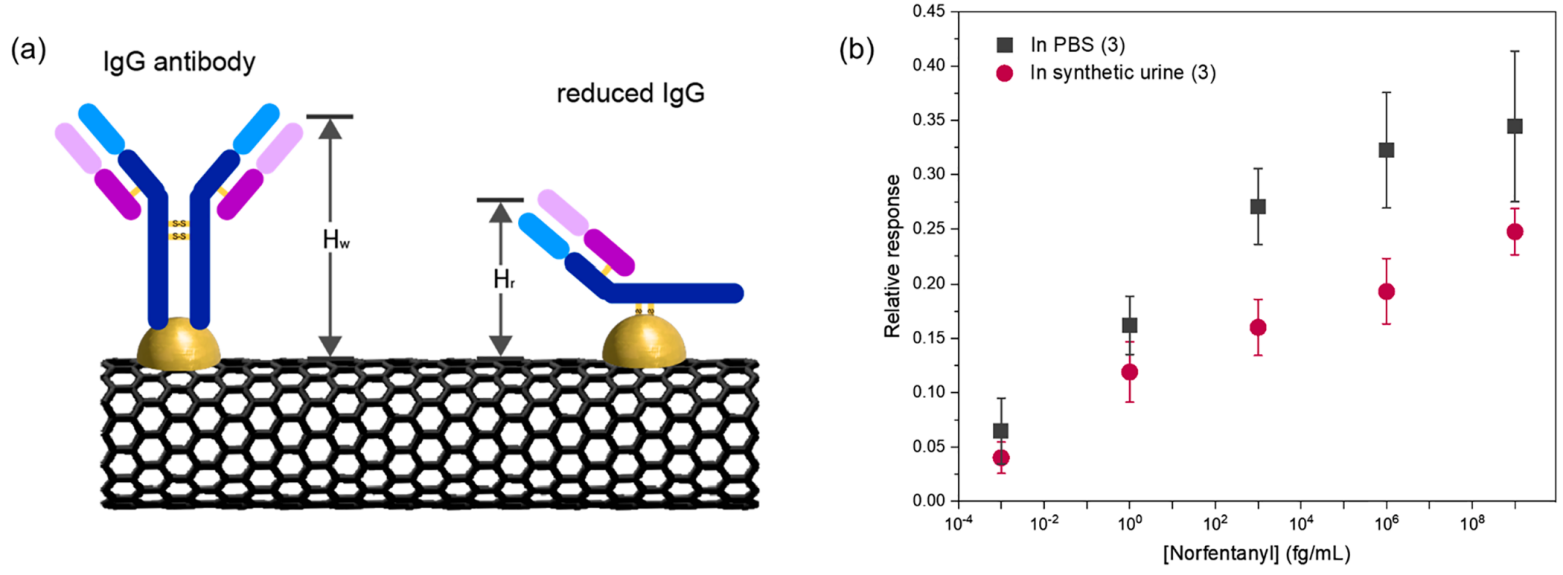
Norfentanyl sensing using reduced norfentanyl antibody-functionalized sc-SWCNT FET biosensors. (a) Binding of whole and reduced IgG antibodies on AuNPs. (b) Norfentanyl sensing in PBS and synthetic urine using reduced norfentanyl ab-Au-sc-SWCNT devices.

The sensitivity of FET biosensors is limited by the Debye screening effect.^46,47^ To further improve the sensitivity of the norfentanyl FET biosensor without compromising the antigen–antibody interaction, one strategy is to reduce the size of the biorecognition elements.^48−50^ Here, we used a mild reducing reagent that cleaves the disulfide bridges in the hinge region of an IgG antibody to produce a “reduced” norfentanyl antibody with a free thiol group while leaving the binding site intact. We hypothesized that by employing the reduced antibody, the orientation of the antibody on the sc-SWCNT surface can be better controlled and the antigen–antibody binding occurs closer to the sensor surface, thus improving the sensitivity (Figure 5a).

The binding between the reduced antibody and the AuNPs was analyzed by XPS. Peaks corresponding to carbon (C), nitrogen (N), oxygen (O), gold (Au), and silicon (Si) can be found on the XPS survey spectrum, confirming the presence of the reduced antibodies on Au-sc-SWCNTs (Figure S9a). Although no obvious sulfur (S) peak is shown in the survey spectrum, peaks associated with Au(I) appear in high-resolution XPS spectra of Au 4f of the device (Figure S9b). We therefore attributed the peaks to the covalent bonding between AuNPs and the free thiol terminals created by reducing the antibody,^51,52^ which confers the control over the orientation of the antibodies on the sensor surface. AFM characterization of the AuNP-sc-SWCNTs before and after the incorporation of reduced antibody reveals an average of 5.02 nm increase in the height of the nanoparticles (Figure S9c, d), significantly lower than the 10.6 nm increase by attaching whole antibody. This result provides further evidence that by employing reduced antibodies, the antigen–antibody interaction can be brought closer to the sensing surface.

As expected, a calibration sensitivity obtained from the reduced antibody-functionalized sc-SWCNT FET devices was 0.039, which is 69.6% higher than those of devices functionalized with whole antibody. This result supports our hypothesis that the reduced distance between the binding site and the sensor surface mitigates the Debye screening effect and enhances the sensitivity of the sensor. An increasing trend in the relative response, however, is observed, which contrasts with the decreasing trend for whole antibody-functionalized AuNP-sc-SWCNT FET devices (Figure 5b). One possible explanation to the opposite sensing behavior is that, by reducing the proximity of the binding sites to the AuNP surface, the binding between the norfentanyl, which relies mainly on the hydrogen−π interaction and cationic−π interaction, reduces the local electron density on the surface of AuNPs,^53^ thus increasing the work function of AuNPs, and consequently increase the conductance of the channels.

In nondiluted synthetic urine, the sensing capability of the reduced antibody-functionalized AuNP-sc-SWCNT FET biosensor for norfentanyl is preserved. A decrease in the calibration sensitivity of the devices is observed when compared to the sensitivity in PBS, which is due to the interferences in synthetic urine that induce negative relative responses of the devices.

In order to enhance the practicality of our developed norfentanyl sensor, we implemented our sensor fabrication technique on commercially available electrodes for better integration with a portable potentiostat. First, sc-SWCNTs were drop-cast on an interdigitated gold electrode on a glass substrate with 10 μm channels (G-IDEAU 10), and norfentanyl antibodies were attached to the CNTs via direct coupling. The selection of the electrode was based on its similarity in sensor geometry to the sensors fabricated in the laboratory (Figure S10a). The sensor performance was evaluated using the calibration curve based on the relative current change at −0.1 *V*_g_. The calibration sensitivity was then determined to be 0.063, which is comparable to the laboratory-fabricated sensors (Figure S10b).

**Figure 6:**
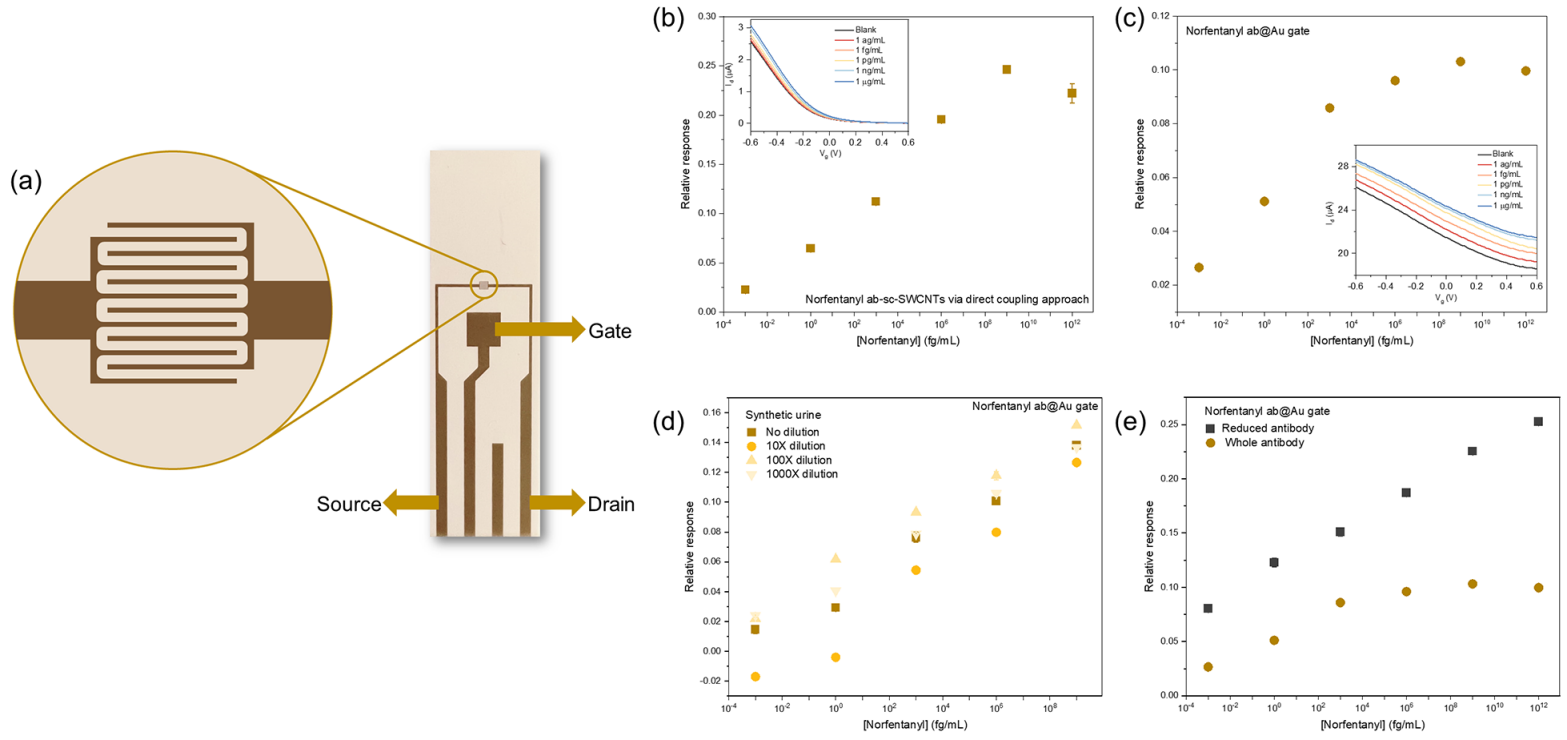
Norfentanyl sensing with flexible gold FETs with a coplanar gate. (a) Optical image of a flexible gold FET with a coplanar gate (AUFET). The zoom-in view is an illustration of interdigitated electrodes. (b) Norfentanyl sensing using AUFET by functionalizing the sc-SWCNTs with norfentanyl antibody via the direct coupling approach. (c) Norfentanyl sensing using AUFET by attaching the norfentanyl antibodies on the Au gate (norfentanyl ab@Au gate). (d) Norfentanyl sensing in synthetic urine using norfetanyl ab@Au gate AUFET. (e) Comparison of norfentanyl sensing performances between whole antibody and reduced antibody functionalized AUFET.

One limitation of G-IDEAU 10 is the requirement of a separate gate electrode, which hinders the portability of the sensor. We therefore applied our sensor fabrication on a flexible gold FET with a coplanar gate (AUFET). The patterned gold gate on the AUFET eliminates the need for a separate gate electrode and fixes the distance between the gate electrode and the semiconducting channels, providing a more controlled sensor configuration (Figure 6a). As a proof of concept, a similar sensor configuration was implemented on the AUFET as previously mentioned. Specifically, sc-SWCNTs were deposited between the IDEs by drop-casting to ensure good conductivity, and norfentanyl antibody was first attached to the sensors via direct coupling. The sensing result in norfentanyl calibration samples exhibits similar sensing behavior with sensors fabricated on the Si chip and good sensing capability for norfentanyl (Figure 6b). However, the small IDE area on the AUFET limits the deposition and functionalization of the sc-SWCNTs, resulting in lower reproducibility of the sensors.

We then took advantage of the gold gate for immobilization of the biorecognition elements of the sensor. By anchoring the antibodies on the gold gate, the binding between the analyte and the antibody alters the capacitance at the gate/electrolyte interface instead of direct modulation on the semiconducting channel.^54−57^ The gold gate, which is 3 × 3 mm in size, provides a large surface area for spontaneous binding of norfentanyl antibodies. Upon addition of norfentanyl, the conductance of the device increases. The sensor calibration made by plotting the relative responses at applied gate voltage of −0.5 V against norfentanyl concentration validates the norfentanyl sensing effect of the AUFET sensor (Figure 6c). Moreover, when tested in synthetic urine, the AUFET devices demonstrated consistent sensing performance regardless of the extent of dilution of synthetic urine, indicative of good reliability for quantitative detection of norfentanyl in a complex matrix (Figure 6d).

Switching the biorecognition element from whole antibody to reduced antibody further improved the sensitivity of the norfentanyl sensor (Figure 6e). The smaller size of the reduced antibody, as well as the more controlled orientation on the gold surface, yields more available binding sites for norfentanyl on the sensing surface. These results suggest the great potential held by antibody-functionalized sc-SWCNT FET biosensors for the development of portable and reliable biosensors for detection of fentanyl exposure.

## Conclusion

Here, we present an sc-SWCNT-based FET biosensor functionalized with norfentanyl antibody for the sensitive detection of norfentanyl, the primary inactive metabolite of fentanyl. Sc-SWCNTs provide a versatile platform for chemical functionalization. FET sensors adopting both the direct coupling approach and AuNP approach for the attachment of norfentanyl antibody demonstrated outstanding sensing capabilities for the detection of norfentanyl, reaching a limit of detection at the fg/mL level. To further optimize the sensing matrix, we decreased the size of norfentanyl antibody and utilized the free thiol groups on the half antibody fragment for the oriented attachment on AuNP-SWCNTs. By reduction of the distance between the binding sites and the sensor surface, the calibration sensitivity of the biosensor was enhanced by 69.6%. Furthermore, we successfully applied our sensor fabrication with a flexible FET electrode with a portable sensing setup, showing great potential for developing a portable device for on-site detection of fentanyl exposure with improved sensitivity.

Overall, the development of effective biosensors for the detection of opioids and their metabolites is crucial for the monitoring of opioid abuse and the management of opioid-related health issues. We also envision that the norfentanyl antibody-functionalized sc-SWCNT-based FET biosensors that we report in this work provide a platform technique for multiplexed sensing for other opioid byproducts to help fight the opioid crisis.

## Methods

### Device Fabrication

The 2.6 × 2.6 mm sensor chip was fabricated on a Si/SiO_2_ substrate and has 8 sensing devices with interdigitated electrodes (IDEs). The IDEs were patterned on the substrate using photolithography, forming 10 μm channels. Semiconducting single-walled carbon nanotubes (IsoSol-S100, Raymor Industries Inc.) were prepared at 0.02 mg/mL in toluene and deposited between gold electrodes via dielectrophoresis (DEP) with an ac frequency of 100 kHz, applied bias voltage of 10 V, and bias duration of 120 s. The devices were annealed at 200 °C for 1 h before use.

For FET devices fabricated via the direct coupling approach, the sc-SWCNTs were first incubated in a 50 mM/50 mM 1-ethyl-3-(3-(dimethylamino)propyl)carbodiimide (EDC)/*N*-hydroxysulfosuccinimide (sulfo-NHS) solution for 30 min to activate the carboxylic acid groups. Norfentanyl antibody (10 μL, 117 μg/mL in PBS buffer) was then introduced on the sc-SWCNTs surface directly after activation and incubated overnight at 4 °C.

For FET devices fabricated via the gold nanoparticle (AuNP) approach, gold nanoparticles were deposited on sc-SWCNTs via bulk electrolysis using a CH Instruments electrochemical analyzer in a three-electrode setup (1 M Ag/AgCl reference electrode, Pt counter electrode, and IDEs as working electrodes) from a HAuCl_4_ solution (1 mM in 0.1 M HCl). The deposition voltage was set at −0.2 V and applied for 30 s. Norfentanyl antibody solution was then immobilized on the device surface by an incubating overnight at 4 °C.

After the attachment of norfentanyl antibody, a blocking buffer (0.1% Tween 20 and 4% poly(ethylene glycol) in PBS) was applied to the device surface to block unreacted surfaces.

### Scanning Electron Microscopy (SEM)

Scanning electron microscopy was performed on a Si/SiO_2_ chip using a ZEISS Sigma 500 VP instrument.

### Atomic Force Microscopy (AFM)

AFM data were collected using a Bruker multimode 8 AFM system with a Veeco Nanoscope IIIa controller in tapping mode. AFM image and height profiles were processed and obtained in Gwyddion.

### X-ray Photoelectron Spectroscopy (XPS)

X-ray photoelectron spectroscopy data was generated on a Thermo ESCALAB 250 Xi XPS instrument using monochromated Al Kα X-rays as the source. A 650 μm spot size was used, and the samples were charge compensated by using an electron flood gun.

### Raman Spectroscopy

Raman characterization of the devices was performed by using a XplorA Raman-AFM/TERS system. Radial breathing mode (RBM) region was recorded using a 785 nm (100 mW) excitation laser operating at 1% power. D and G peak regions were recorded using 638 nm (24 mW) excitation laser operating at 1% power.

### Cyclic Voltammetry (CV)

Norfentanyl sensing using sc-SWCNT-based FET biosensors via cyclic voltammetry was conducted by using a CH Instruments electrochemical analyzer. A three-electrode configuration was used, where sc-SWCNT acted as the working electrode, Ag/AgCl electrode was used as the reference electrode, and Pt wire was used as the auxiliary electrode. The CV experiments were performed by sweeping the voltage from −0.6 to +0.6 V for 10 cycles with a scan rate of 20 mV/s.

### FET Measurements

A Metrohm DropSens μStat-i 400 potentiostat was used for all of the FET measurements. FET transfer characteristics were measured by employing a liquid-gated FET device configuration. A 1 M Ag/AgCl reference electrode was used as the gate electrode. Source-drain current (*I*_d_) was collected by sweeping the gate voltage (*V*_g_) from +0.6 V to −0.6 V while keeping the source-drain voltage at 50 mV. The gating media was 0.001× PBS.

A series of norfentanyl solutions were prepared from 1.0 ag/mL to 1.0 μg/mL. For calibration samples, the solutions were prepared in 1× phosphate buffered saline (PBS). For synthetic urine samples, 1× synthetic urine was first prepared according to Table S1. The norfentanyl solutions were prepared in 1× synthetic urine and followed by 1:10, 1:100, and 1:1000 dilution with 1× PBS. All norfentanyl samples were tested from the lowest to the highest concentrations.

For each device, FET transfer characteristics were first collected in a blank sample, which would be used as a baseline. Next, 10 μL of each norfentanyl sample was added to the surface of the device and the mixture incubated for 10 min. After the incubation, the device was rinsed with nanopure water to remove the unbound sample, and FET transfer characteristics were collected in the gating medium in order to keep the same ionic strength for all FET measurements.

The relative response (*R*) of each FET device was calculated as *R* = Δ*I*/*I*_0_ at *V*_g_ = −0.5 V, where Δ*I* = *I*_d_ – *I*_0_, and *I*_0_ is the drain current in blank sample (baseline) before analyte exposure at applied gate voltage of −0.5 V. The calibration curve was plotted by reporting the averaged relative conductance of all devices tested with standard error as error bars at each concentration. The number of devices (*n*) tested for each experiment is specified in the figure. Calibration sensitivity was defined as the slope of the linear region on the calibration curve. The linear region was located by fitting the calibration curve using a Logistic model.

The limit of detection was calculated using the formula LOD = 10^3δ/S^, where δ denotes the standard deviation of the blank test, and S denotes the slope of the linear region of the calibration plot. For blank test, both types of sensors were incubated with the blank sample (i.e., 1× PBS) and taken FET measurements for 5 times after the initial blank measurement. The relative change at each test was calculated, and δ was determined from the 5 tests.

### Reduction of Norfentanyl Antibody

The reaction buffer was prepared by adding 10 mM ethylenediaminetetraacetic acid (EDTA) to 1× PBS. Six milligrams of 2-mercaptoethylamine·HCl (2-MEA) was dissolved in 100 μL of Reaction Buffer, and then 5 μL of this 2-MEA solution was immediately added to 50 μL of norfentanyl antibody solution (1.03 mg/mL) in PBS. The reaction mixture was kept in an incubator at 37 °C for 90 min. After the reaction, buffer exchange was performed using a desalting column to remove 2-MEA from the reduced antibody. The final solution with the reduced norfentanyl antibody was aliquoted and frozen for further use.

## Supporting Information Available

Table of synthetic urine components, additional norfentanyl antibody-functionalized sc-SWCNT FET device characterizations, cyclic voltammograms, sensor responses to synthetic urine, AFM and XPS characterizations of the reduced antibody-functionalized biosensors (PDF)
