# Supplementary material for: An Ultrasensitive Norfentanyl Sensor Based on a Carbon Nanotube-Based Field-Effect Transistor for the Detection of Fentanyl Exposure
Source: ACS Appl Mater Interfaces. 2023 Jul 31;15(31):37784–93. doi: 10.1021/acsami.3c05958 (PMC10416144; doi:10.1021/acsami.3c05958)
Supplement: Supplementary file 1 — am3c05958_si_001.pdf [file am3c05958_si_001.pdf]

Supporting Information

An Ultrasensitive Norfentanyl Sensor Based on a Carbon Nanotube-Based Field-Effect Transistor for the Detection of Fentanyl Exposure

Wenting Shao,[†] *Zidao Zeng*[†] *and Alexander Star*[*, †, ‡]

† Department of Chemistry, University of Pittsburgh, Pittsburgh, Pennsylvania 15260, United States

‡ Department of Bioengineering, University of Pittsburgh, Pittsburgh, Pennsylvania 15261, United States

*Corresponding author. Email: astar@pitt.edu

TABLE OF CONTENTS

Table S1. Synthetic urine composition ·· 3

Figure S1. XPS characterization of norfentanyl antibody-functionalized sc-SWCNT FET devices using the direct coupling approach ·· 4

Figure S2. Norfentanyl sensing using cyclic voltammetry ································ 5

Figure S3. Comparison between Keithley 2400 sourcemeters and Metrohm DropSens μStat-i 400 potentiostat ··· 6

Figure S4. Comparison of sensor responses in 0.001× PBS and norfentanyl samples ············ 7

Figure S5. Calibration plots for determination of calibration sensitivity and limit of detection ··· 8

Figure S6. Specificity study ··· 9

Figure S7. Sensor responses to synthetic urine ·· 10

Figure S8. Comparison of calibration sensitivity in PBS and 1000-fold diluted synthetic urine 11

Figure S9. Characterizations of the reduced norfentanyl antibody-functionalized Au-sc-SWCNT FET devices ··· 12

Figure S10. Norfentanyl sensing with an interdigitated gold electrode on a glass substrate ····· 13

Table S1. Synthetic urine composition.

Formula	Molarity (mM)	Quantity (g/100mL)
CH_4N_2O	249.750	1.5000
KCl	30.953	0.2308
NaCl	30.053	0.1756
NH_4Cl	23.667	0.1266
$NaH_2PO_4{\cdot}2H_2O$	18.667	0.2912
Na_2SO_4	11.965	0.1700
$C_4H_7N_3O$	7.791	0.0881
$Na_2HPO_4{\cdot}2H_2O$	4.667	0.0831
$MgSO_4{\cdot}7H_2O$	4.389	0.1082
$Na_3C_6H_5O_7{\cdot}2H_2O$	2.450	0.0720
$CaCl_2$	1.663	0.0185
$C_5H_4O_4N_3$	1.487	0.0250
$K_2C_2O_4{\cdot}H_2O$	0.19	0.0035

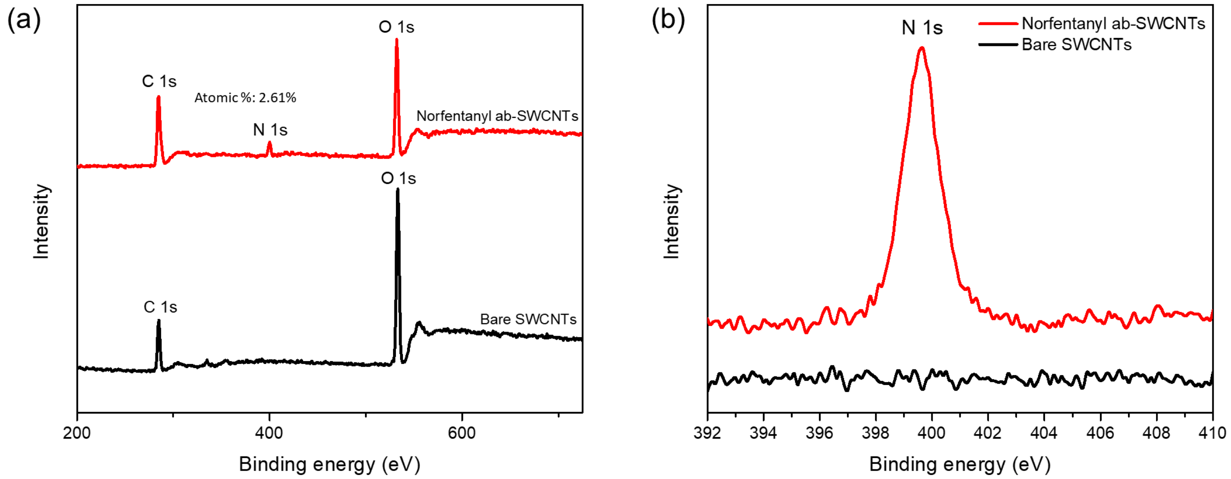

Figure S1. XPS characterization of norfentanyl antibody-functionalized sc-SWCNT FET devices using the direct coupling approach. (a) XPS survey of the FET device before and after the functionalization of norfentanyl antibody. (b) High-resolution XPS spectra of N 1s of the FET device before and after the functionalization of norfentanyl antibody. The appearance of the N peak indicated the successful attachment of norfentanyl antibodies.

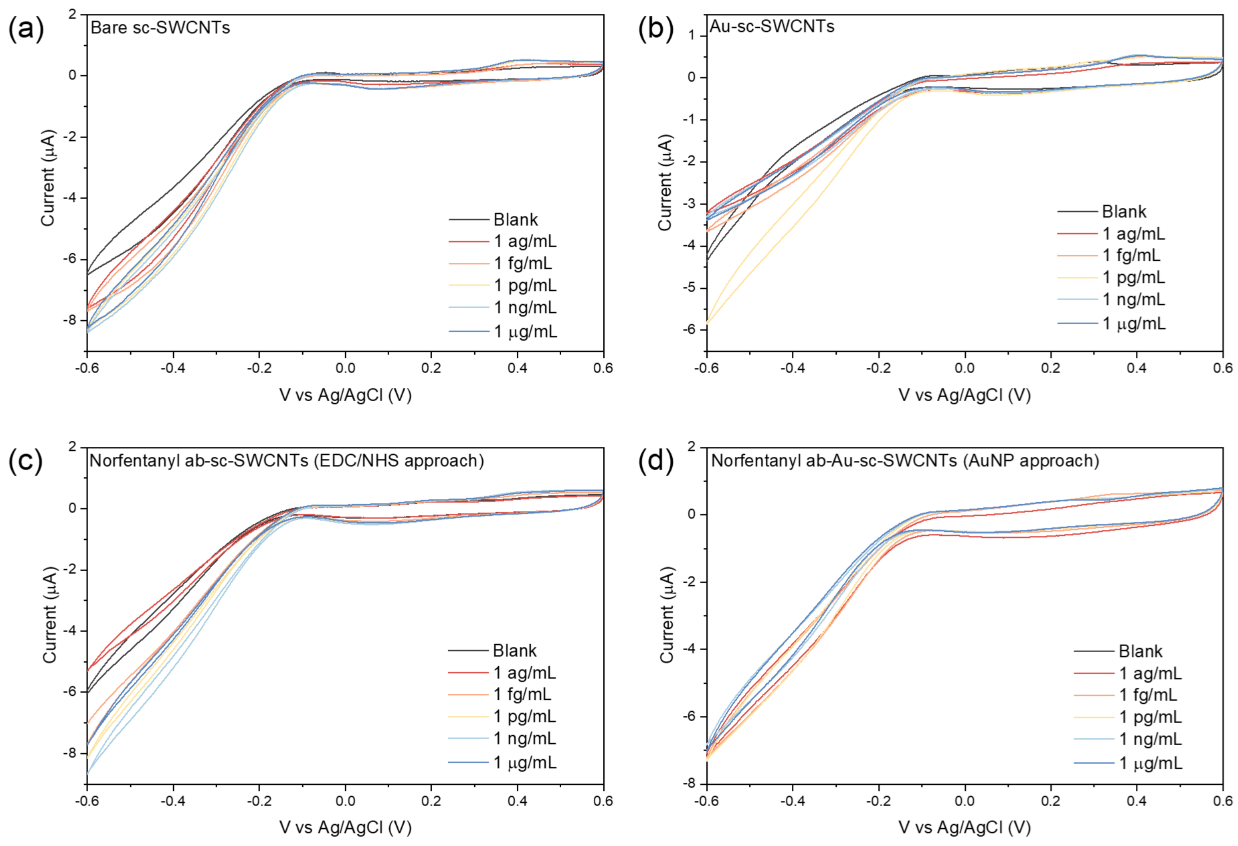

Figure S2. Norfentanyl sensing using cyclic voltammetry. (a) Bare sc-SWCNTs device. (b) Au-sc-SWCNTs device. (c) Norfentanyl ab-sc-SWCNTs device. (d) Norfentanyl ab-Au-sc-SWCNTs device.

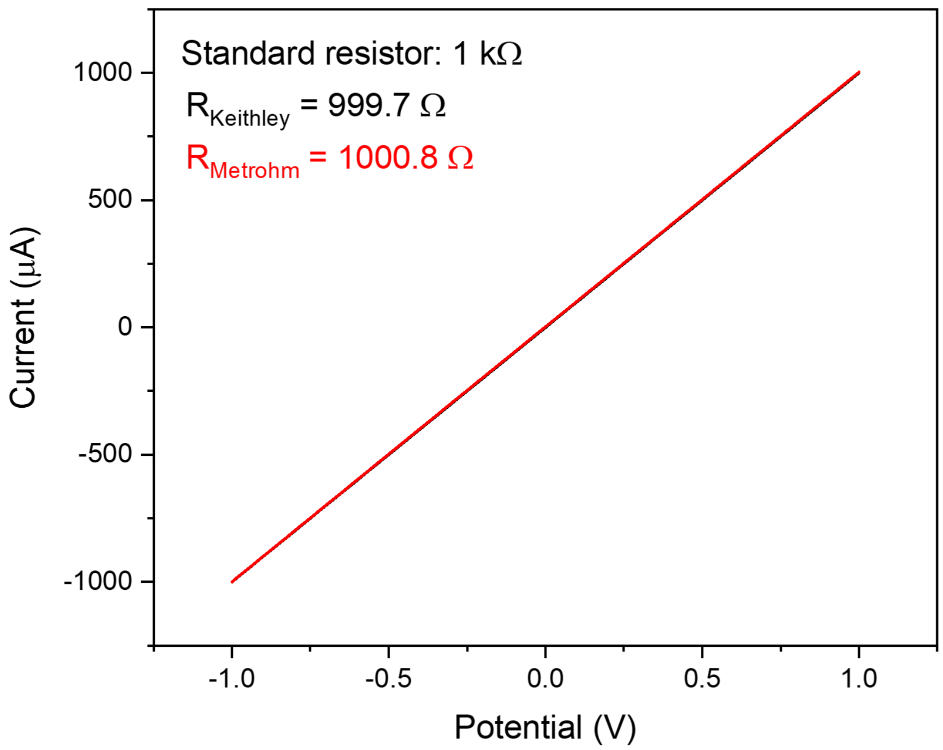

Figure S3. Comparison between Keithley 2400 sourcemeters and Metrohm DropSens µStat-i 400 potentiostat.

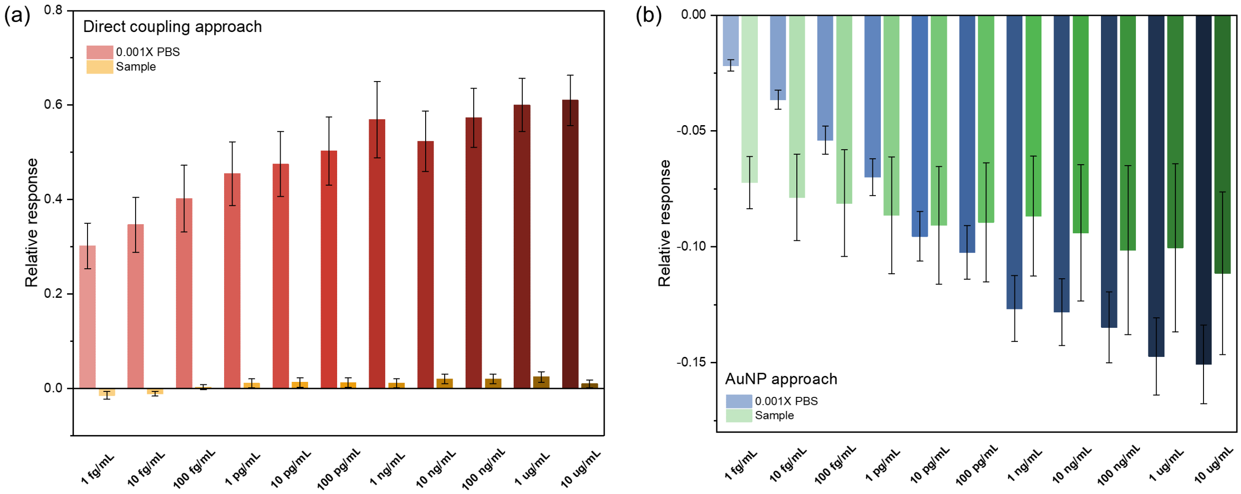

Figure S4. Comparison of sensor responses in 0.001× PBS and norfentanyl calibration samples. (a) Relative responses of devices adopting the direct coupling approach with increasing norfentanyl concentration. (b) Relative responses of devices adopting the AuNP approach with increasing norfentanyl concentration. For both types of sensors, the sensitivities were significantly improved when testing in 0.001× PBS.

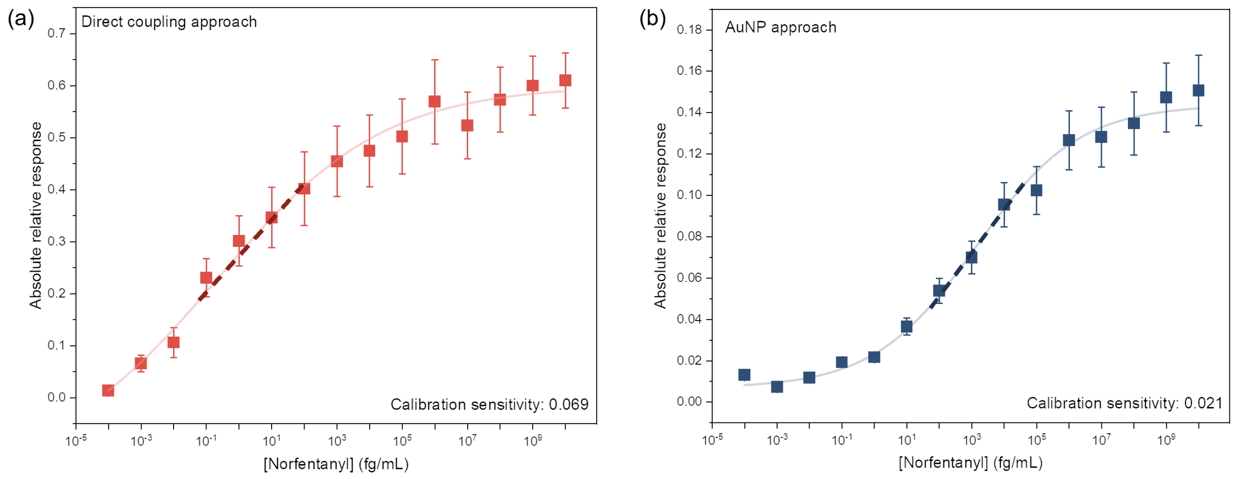

Figure S5. Calibration plots for determination of calibration sensitivity and limit of detection. (a) Calibration curve for devices fabricated using the direct coupling approach. (b) Calibration curve for devices fabricated using AuNP approach.

Both calibration curves were fitted using a Logistic model, and the linear regions were indicated by the dotted lines. The calibration sensitivity for each type of sensor was determined by the slope of the linear region. For devices fabricated using the direct coupling approach, the calibration sensitivity was 0.069. For devices fabricated using AuNP approach, the calibration sensitivity was 0.021.

The limit of detection (LOD) was calculated using the formula LOD = $10^{(3\delta/S)}$, where δ denotes the standard deviation of the blank test and S denotes the slope of the linear region of the calibration plot. From the blank test, δ was found to be 0.007 for devices fabricated using the direct coupling approach and 0.004 for devices fabricated using AuNP approach. Therefore, LOD was calculated to be 2.0 fg /mL and 3.7 fg/mL respectively.

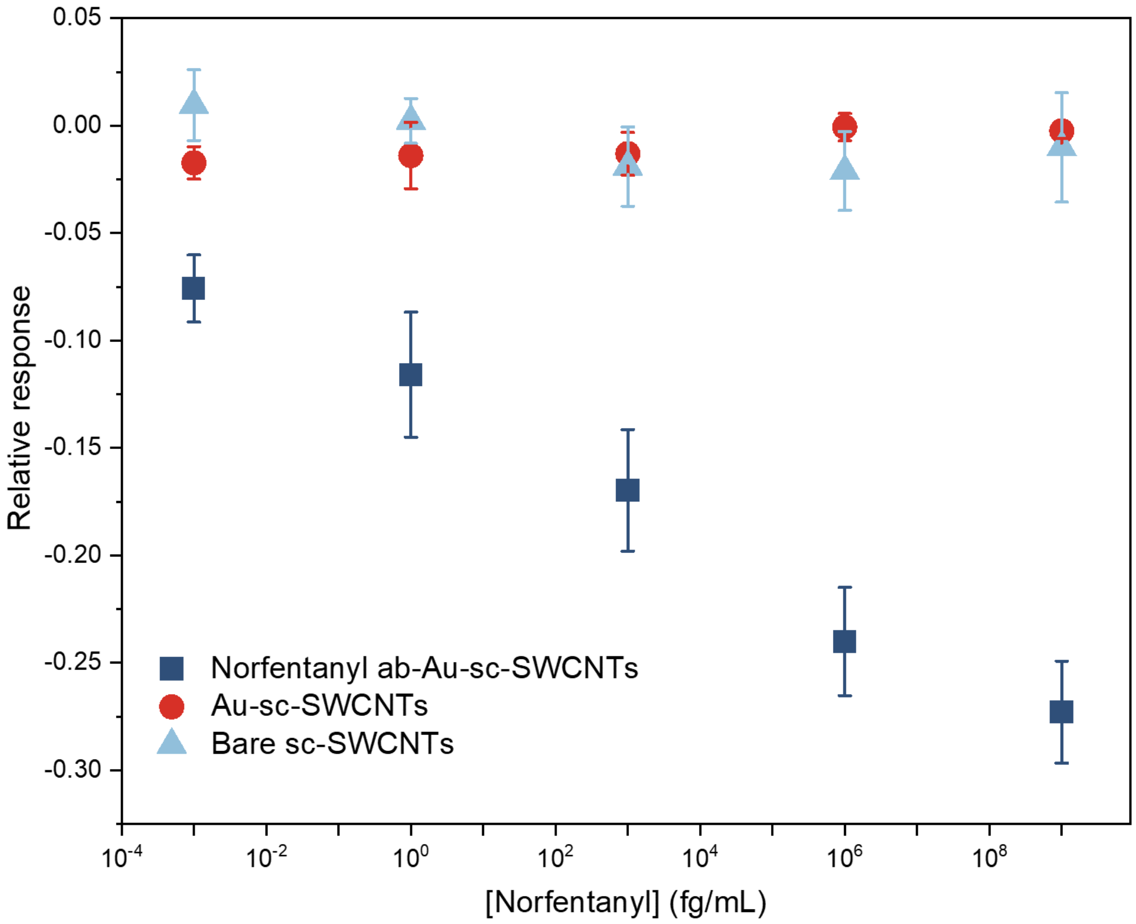

Figure S6. Specificity study. Norfentanyl sensing experiments were performed on bare sc-SWCNTs devices and Au-sc-SWCNTs. The results suggested that the devices did not respond to norfentanyl when norfentanyl antibody was absent.

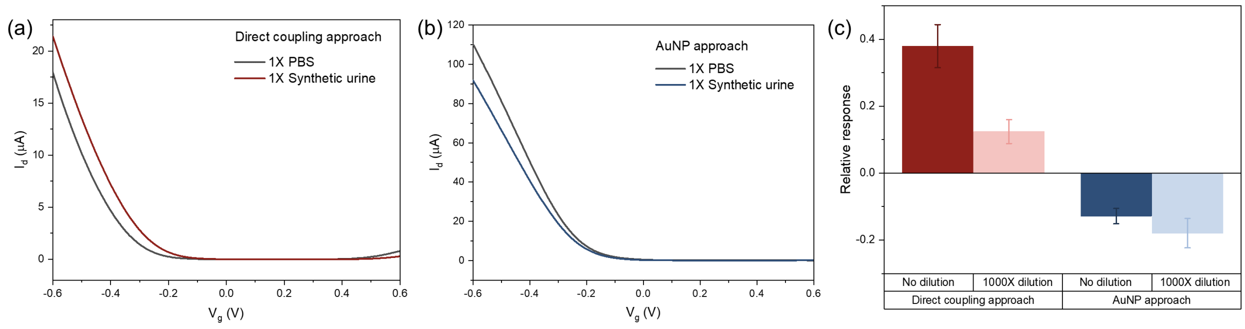

Figure S7. Sensor responses to synthetic urine. (a) FET characteristics of devices fabricated using the direct coupling approach in 1X PBS and 1X synthetic urine. (b) FET characteristics of devices fabricated using the AuNP approach in 1X PBS and 1X synthetic urine. (c) Relative responses of both types of devices in non-diluted and 1000-fold diluted synthetic urine.

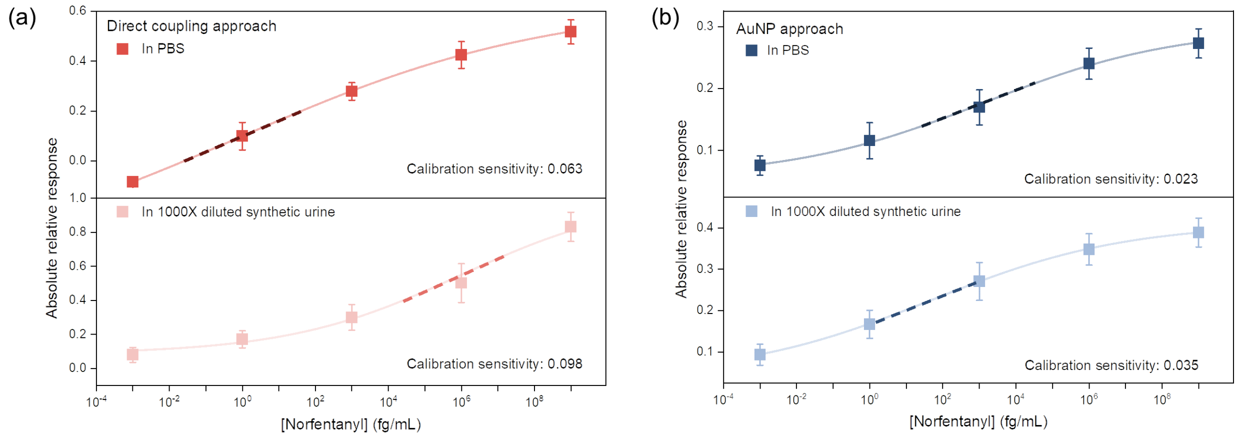

Figure S8. Comparison of calibration sensitivity in PBS and 1000-fold diluted synthetic urine. (a) Devices fabricated using the direct coupling approach. (b) Devices fabricated using the AuNP approach. Solid lines are fitted using a Logistic model. Dashed lines indicate the linear regions on the calibration curves.

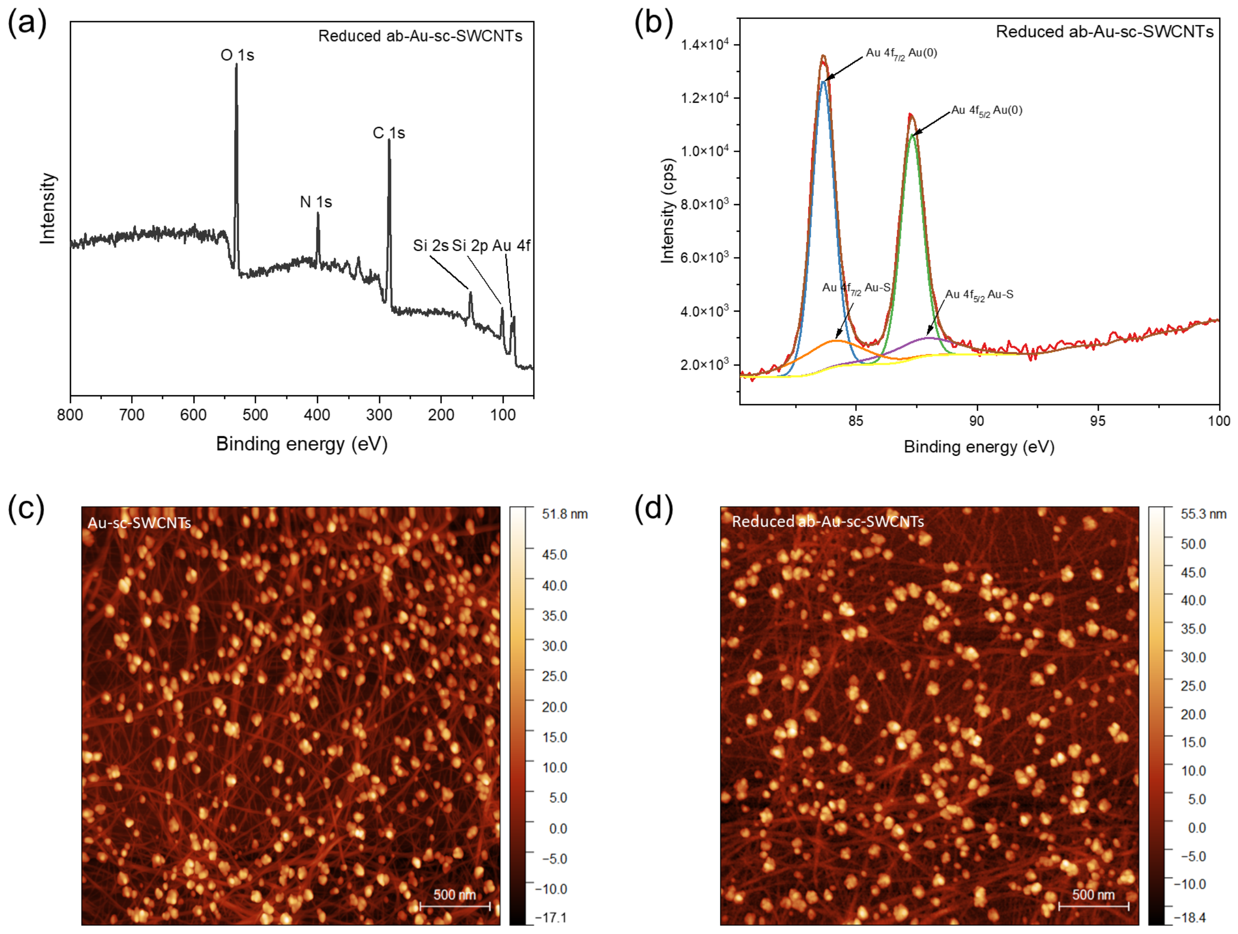

Figure S9. Characterizations of the reduced norfentanyl antibody-functionalized Au-sc-SWCNT FET devices. (a) XPS survey and (b) High-resolution XPS spectra of Au 4f of the reduced ab-Au-sc-SWCNTs FET device. (c) AFM image of bare AuNP-sc-SWCNT FET device. (d) AFM image of AuNP-sc-SWCNT FET device after immobilization of reduced norfentanyl antibody (reduced ab-Au-sc-SWCNTs).

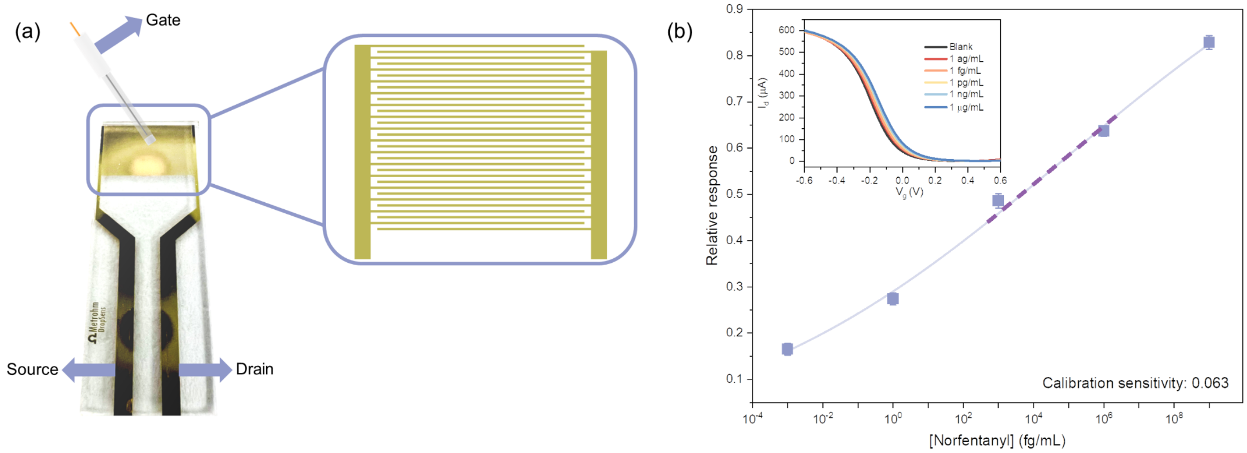

Figure S10. Norfentanyl sensing with an interdigitated gold electrode on a glass substrate (G-IDEAU 10). (a) Optical image of the G-IDEAU 10. The zoom-in view is an illustration of interdigitated electrodes. (b) Norfentanyl sensing performance of G-IDEAU 10. The calibration curve was established using relative current change at –0.1 Vg. Inset shows FET transfer characteristics upon adding increasing concentrations of norfentanyl.
